# Supplementary figures and images for: Designing an Electronic Patient Reported Outcomes Information Infrastructure Supported by the RE-AIM Implementation Framework
Source: medRxiv. 2025 Apr 1:2025.03.31.25324980. Preprint. [Version 1] doi: 10.1101/2025.03.31.25324980 (PMC11998853; doi:10.1101/2025.03.31.25324980)

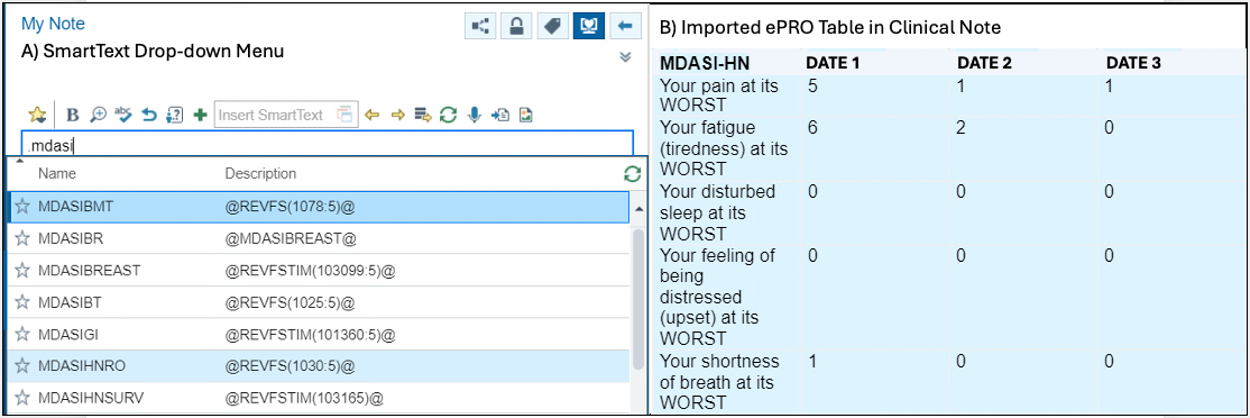

Supplement: Supplement 1 — Supplemental Figure 1. SmartText Example. [file media-1.jpg]

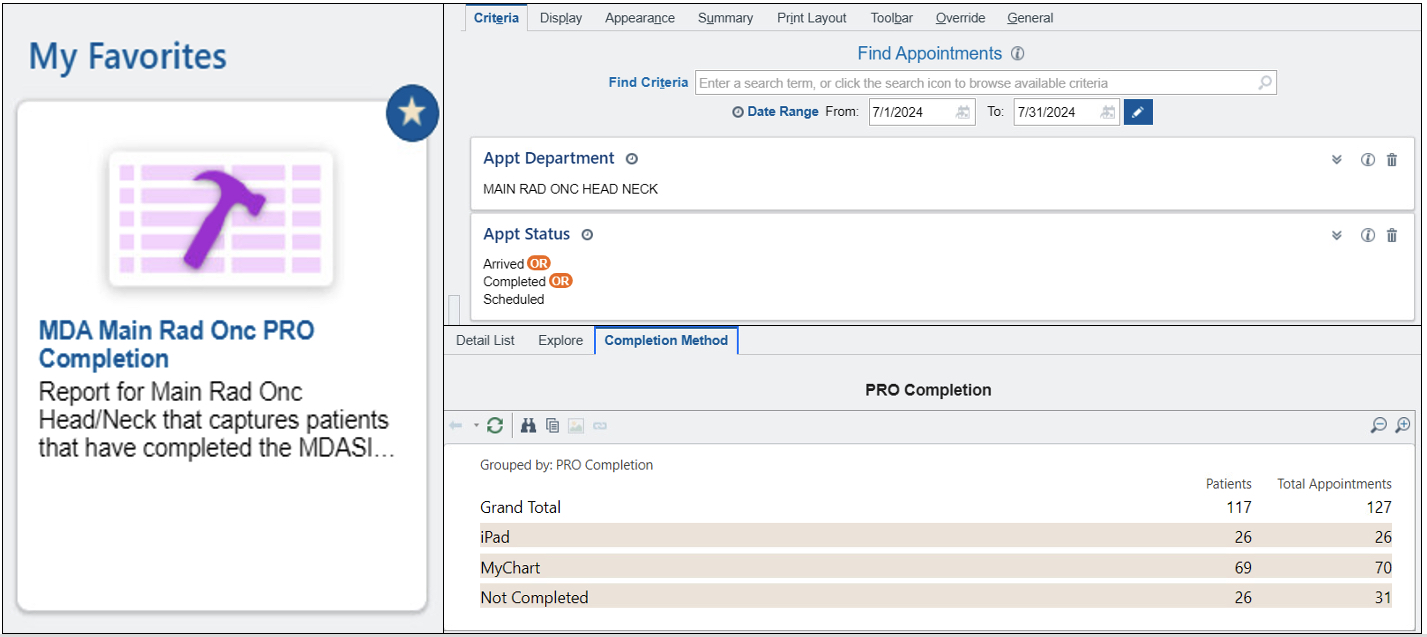

Supplement: Supplement 2 — Supplemental Figure 2. EHR Reporting Workbench PRO Completion Report. [file media-2.jpg]
